# Brain‐derived neurotrophic factor prevents dendritic retraction of adult mouse retinal ganglion cells

**DOI:** 10.1111/ejn.13295

**Published:** 2016-07-19

**Authors:** Kate E. Binley, Wai S. Ng, Yves‐Alain Barde, Bing Song, James E. Morgan

**Affiliations:** ^1^ School of Optometry and Vision SciencesCardiff UniversityMaindy RoadCardiffCF24 4HQUK; ^2^ School of BiosciencesSir Martin Evans BuildingCardiff UniversityCardiffUK; ^3^ School of DentistryCardiff UniversityHeath ParkCardiffUK

**Keywords:** neurodegeneration, neuron labelling, neuroprotection, Sholl analysis

## Abstract

We used cultured adult mouse retinae as a model system to follow and quantify the retraction of dendrites using diolistic labelling of retinal ganglion cells (RGCs) following explantation. Cell death was monitored in parallel by nuclear staining as ‘labelling’ with RGC and apoptotic markers was inconsistent and exceedingly difficult to quantify reliably. Nuclear staining allowed us to delineate a lengthy time window during which dendrite retraction can be monitored in the absence of RGC death. The addition of brain‐derived neurotrophic factor (BDNF) produced a marked reduction in dendritic degeneration, even when application was delayed for 3 days after retinal explantation. These results suggest that the delayed addition of trophic factors may be functionally beneficial before the loss of cell bodies in the course of conditions such as glaucoma.

## Introduction

The adult murine retina provides an attractive model to study detailed structural alterations of neurons following axotomy (Johnson & Martin, 2008; Bull *et al*., 2011; Guerin *et al*., 2011; Wood *et al*., 2011; Denk *et al*., 2015; White *et al*., 2015). While the loss of retinal ganglion cells (RGCs) has typically been used as the primary read‐out in this preparation (Bull *et al*., 2011), fewer studies address the changes that might occur in the dendrites of axotomized RGCs prior to cell loss (see Discussion for a very recent example, Johnson *et al*., 2016). Yet dendritic atrophy following axotomy may be of considerable functional relevance while also offering a therapeutic opportunity in conditions such as glaucoma (Weber *et al*., 1998; Morgan, 2012; El‐Danaf & Huberman, 2015). In principle, dendritic arbourization can be readily quantified by analytical tools, such as Sholl analysis (Sholl, 1953), in which proximal dendrite loss is reflected by a lowered peak amplitude and reduced terminal branching density is indicated by a leftward shift in the profile. Sholl analysis has been further developed with the introduction of the notion of a ‘branching index’ (Garcia‐Segura & Perez‐Marquez, 2014). While Sholl analysis is as such well‐established and used extensively to quantify dendritic arbourization in numerous central nervous system (CNS) structures, the procedures allowing unbiased and sparse labelling of individual cells are less straightforward. In particular, the RGC morphology varies greatly between cell types in the rodent eye, reflecting the large number of individual cell types (Sun *et al*., 2002; Coombs *et al*., 2006). In view of this, random labelling of RGCs would be desirable to minimize bias. Sparse labelling using transgenic animals is frequently used to analyse dendritic arbours in the CNS including those of RGCs, typically using the Thy1 promoter coupled to genetically encoded fluorescent reporters. While elegant and convenient, this approach is not without problems given observations by us and others that the Thy1 promoter may be up or downregulated following axotomy (Lee *et al*., 1998; Schlamp *et al*., 2001; Huang *et al*., 2006; Astafurov *et al*., 2014). As the mechanisms underlying sparse expression of the Thy1 promoter in transgenic animals are still unknown, it is also unclear whether RGC labelling is truly random or not. In this study, we use an acute labelling technique designated diolistics (Gan *et al*., 2000) to determine the time course of neuronal dendritic atrophy following RGC axotomy. The use of this technique revealed that dendritic alterations occur within hours following axotomy and precede by days the loss of RGCs, thus allowing us to explore the effect of brain‐derived neurotrophic factor (BDNF) on dendrites days before any confounding effects caused by the rescue of RGC cell bodies. This is potentially a significant confounding factor as BDNF has been known for a long time to prevent the death of RGCs in a number of species, both *in vitro* and *in vivo* after axotomy (Rodriguez‐Tebar *et al*., 1989; Meyer‐Franke *et al*., 1995; Di Polo *et al*., 1998).

## Materials and methods

### Explant preparation and culture

Seventy‐eight adult C57/Bl6 mice aged 1.5–8.5 months of either sex were killed by cervical dislocation in accordance with United Kingdom Home Office regulations. Eyes were immediately enucleated into ice‐cold Hanks balanced Salt Solution (Invitrogen Ltd., Paisley, UK), and the retinas rapidly dissected onto 0.4 μm polytetrafluoroethylene culture inserts (Millipore, Watford, UK), ganglion cell layer (GCL) facing up. Inserts were placed into 35 mm culture dishes (Millipore) containing 1.2 mL Neurobasal‐A culture medium (Invitrogen Ltd.) supplemented with 1% penicillin‐streptomycin (Invitrogen Ltd.), 0.8 mm L‐glutamine (Invitrogen Ltd.), 1% N2 supplement (Invitrogen Ltd.) and 2% B27 supplement (Invitrogen Ltd.). Explants were cultured at 37 °C, 5% CO_2_ for up to 14 days. For culture periods exceeding 1 day, the medium was replaced daily to prevent accumulation of toxins.

### Nuclear staining

At the end of the culture period 18 explants (*n* = 15 mice of both sex, aged 2–7 months) were fixed (4% paraformaldehyde, 4 h), cryoprotected (30% sucrose, overnight) and frozen in optimal cutting temperature compound. Sagittal sections (10–14 μm) were cut using a cryostat (Leica CM 3050S), nuclear stained (TO‐PRO‐3, 10 min) and coverslipped with Prolong Gold anti‐fade reagent (Invitrogen Ltd.). Sections were imaged at 20× by confocal microscopy (Zeiss, lsm 510, release version 4.2 SP1) (633 nm laser with 651–704 nm detector). Z‐stacked 1024 × 1024 pixel images were obtained in 1 μm steps and the central 1 μm slice analysed.

### Immunohistochemistry

At the end of the culture period 16 retinas (*n* = 14 mice of both sex, aged 2–7 months) were fixed and cryosectioned (v.s.). Sections were labelled with the RGC markers NeuN, TUJ1 and Thy1.2, and the apoptotic marker active caspase‐3. Antibody working concentrations and sources are given in Table 1. Incubations were at room temperature, unless stated otherwise. Sections were blocked with 5% chicken serum (Invitrogen Ltd., 30 min), then incubated with primary antibody (4 h or overnight at 4 °C). Sections were incubated with secondary antibody (1.5 h), nuclear stained with TO‐PRO‐3 (10 min), and coverslipped with Prolong Gold anti‐fade reagent. Positive controls were anterior sections of sagittally cut mouse brain (NeuN, TUJ1 and Thy1.2) or mouse spleen sections (active caspase‐3). Negative controls were as follows: primary antibody only, secondary antibody only, no antibody. Sections were imaged at 20× by confocal microscopy using separate channels for the fluorophore (488 nm argon laser with 500–530 nm bandpass filter) and nuclear stain (633 nm laser). Z‐stacked 1024 × 1024 pixel images were obtained in 1 μm steps. The central 1 μm slice was analysed. NeuN‐ and TUJ1‐positive cells in the GCL were double stained for nuclear stain and antibody. Mean green channel intensity was calculated with the image processing software fiji (Schindelin *et al*., 2012) as the [mean green channel count for the layer] − [mean green channel count for background outside the region of interest].

**Table 1:** Antibodies and stains used for immunohistochemistry

Primary antibodies					
Specificity	Cells labelled	Source (isotype)	Clone	Company	Working concentration
NeuN	Neurons	Mouse (IgG2a)	Monoclonal	Abcam	5 μg/mL
TUJ1	Neurons	Mouse (IgG2a)	Monoclonal	Covance	5 μg/mL
Thy1.2	RGCs	Rat (IgG2b)	Monoclonal	Abcam	2.5 μg/mL
Active caspase‐3	Apoptotic cells	Rabbit	Polyclonal	Chemicon (Millipore)	10 μg/mL

Secondary antibodies and nuclear stain				
Specificity	Conjugate	Source	Company	Working concentration
Mouse IgG	AlexaFluor 488	Goat	Invitrogen – Molecular Probes Inc.	4 μg/mL
Rat IgG	AlexaFluor 488	Goat	Abcam	4 μg/mL
Rabbit IgG	AlexaFluor 488	Goat	Invitrogen – Molecular Probes Inc.	4 μg/mL
TO‐PRO‐3 iodide 642			Invitrogen – Molecular Probes Inc.	1 μm

### Viability assay

The terminal deoxynucleotidyl transferase dUTP nick‐end labelling (TUNEL) assay (Millipore, UK Ltd.) was used according to the manufacturer's protocol to quantify apoptosis in retinas cultured for up to 14 days. Briefly, frozen sections of 14 explants (*n* = 13 mice of both sex, aged 2–7 months) were digested with proteinase‐K before labelling with TdT end‐labelling cocktail, and then Avidin‐FITC. Sections were counterstained with TO‐PRO‐3, and coverslipped with Prolong Gold Anti‐fade reagent. Positive controls were retinal sections pre‐incubated in proteinase‐K for 1 h. Negative controls were prepared by omission of Avidin‐FITC. Sections were imaged at 20× by confocal microscopy using separate channels for Avidin‐FITC (488 nm argon laser with 500–530 nm bandpass filter) and TO‐PRO‐3 (633 nm laser). Z‐stacked 1024 × 1024 pixel images were obtained and analysed as previously described. Mean green channel intensity was calculated as before.

### Diolistics and Sholl analysis

Fifty explants (*n* = 38 mice of both sex, aged 1.5–5 months) were cultured for up to 3 days. Tungsten particles (200 mg) were coated with 3 mg 1‐1‐dioctadecyl‐3,3,3,3‐tetramethylindocarbocyanine perchlorate (DiI) and 6 mg 3,3‐dioctadecyloxacarbocyanine perchlorate (DiO), and distributed along Tefzel tubing (Bio‐rad, Hertfordshire, UK) using a Tubing Prep Station (Bio‐rad). The tubing was cut into 1.2 cm ‘bullets’ (Gan *et al*., 2000). At the end of the culture period, DiI/DiO‐coated tungsten particles were fired using a Helios gene gun (Bio‐rad) 5 cm from the retinal surface at 120 psi (helium) through a 3 μm polyethylene terephthalate membrane filter (Scientific Laboratory Supplies Ltd., Yorkshire, UK) to prevent clumping of dye particles. Explants were then incubated at 37 °C, 5% CO_2_ for 30 min, followed by fixation with 4% paraformaldehyde (10 min), and nuclear stained with TO‐PRO‐3 (10 min). Finally, explants were coverslipped with Prolong Gold anti‐fade reagent and imaged within 24 h.

Diolistically labelled RGCs were identified as having a soma in the GCL and an axon projecting to the optic nerve. RGCs were imaged at 20× by confocal microscopy using separate channels for each dye (DiI 543 nm laser with 565–615 nm bandpass filter; DiO 488 nm argon laser with 500–530 nm bandpass filter). Z‐stacked 512 × 512 or 1024 × 1024 8‐bit pixel images of the entire cell were obtained in 1 μm steps. Cell eccentricities relative to the optic nerve head were noted using the Stage Controller Stepper (Zeiss). RGC images were imported into fiji and dendritic arbours traced in 3D. Dendritic integrity was quantified by 3D Sholl analysis using the Simple Neurite Tracer plugin (Longair *et al*., 2011) centred on the soma centre with a sphere interval of 10 μm. The area under the Sholl profile was calculated using the trapezoidal model. The branching index was calculated as described by Garcia‐Segura and Perez‐Marquez using the number of dendrite intersections, *I*, at each radial distance from the soma centre, *x* (Garcia‐Segura & Perez‐Marquez, 2014). Briefly, at each radial distance > 0 μm the number of new dendrites was multiplied by the radial distance. The sum equated to the branching index for that cell. Note that this index accounts for branching events only.

$$\text{Branching index}=\sum_{x_{i}}^{x_{\max}}[I_{x}-\left(I_{x-n}\right)]\cdot x$$

where *x*_*i*_ = 10 μm; *x*_max_ = 300 μm; *n *=* *10 μm; [*I*_*x*_ − (*I*_*x* − *n*_)] > 0.

### BDNF treatment

To test a possible role of BDNF in delaying RGC dendropathy, the medium was supplemented with 100 ng/mL *E. coli* recombinant BDNF (Regeneron/Amgen) diluted in 1× PBS containing 0.1% bovine serum albumin (BSA). Ten explants (*n* = 10 mice of both sex, aged 2–5.5 months) were cultured in BDNF‐supplemented medium for 3 days initiated at 0 day. Controls had medium with the same volume aliquot (2 μL/1 mL medium) of vehicle. As before, the medium was replaced daily, along with fresh BDNF (100 ng/mL) or vehicle. At the end of the culture period RGCs were diolistically labelled and analysed by Sholl analysis. Dendritic field area was calculated as the area enclosed by a linear polygon connecting the terminal dendrites. All cells were masked prior to analysis.

### Pan‐caspase inhibition

To assess the role of caspase activation in RGC dendritic retraction, the pan‐caspase inhibitor, Q‐VD (R&D Systems, Abingdon, UK), was applied to nine explants (*n* = 6 mice, female, aged 3–4 months). Q‐VD has been shown to be one of the most effective broad‐spectrum caspase inhibitors and at 100 μm Q‐VD is non‐toxic and highly effective at preventing apoptosis in mouse lymphoblast cells (Caserta *et al*., 2003) and 52.6 μm Q‐VD protects against RGC death in the rat retina (Patil & Sharma, 2004). A topical aliquot (100 μL) of 100 μm Q‐VD, or vehicle (DMSO) as control, was applied daily. After 2 days retinas were labelled (diolistics) and nuclear stained (TO‐PRO‐3). Sholl profiles were derived for labelled cells as described. Cell analysis was undertaken in a masked fashion.

### Delayed BDNF treatment

To test the neuroprotective role of BDNF in a more clinically relevant setting, six explants (*n* = 6 mice, male, aged 8.5 months) were cultured in BDNF‐free medium for 3 days, followed by culture in BDNF‐supplemented medium (100 ng/mL BDNF) for a further 3 days (6 days total). Fresh BDNF was added with each daily medium change. Controls had vehicle only. RGCs were labelled diolistically and analysed in a masked fashion as before.

### Cell sub‐type analysis

To account for cell‐subtype bias, cells were classified as ON, OFF and bistratified according to dendritic termination within the inner plexiform layer (IPL); cells with dendrites terminating within the first 3/5 (~ 9 μm) of the IPL were categorized as ON, those with dendrites terminating in the end 2/5 (~ 6 μm) of the IPL were categorized as OFF. Bistratified cells had dendrites terminating in both sublaminae.

### Statistical analysis

Data processing was carried out using microsoft excel (Office 2013). Statistical analysis was carried out using ibm spss (version 20). Normality of data was tested using the Shapiro–Wilk test. To compare two groups independent samples *t*‐test, pairwise one‐way anova or pairwise Kruskal–Wallis one‐way anova was used. Comparison between multiple groups was made by one‐way anova with TUKEY post hoc, or Mann–Whitney with Bonferroni correction. *P *<* *0.05 was the level of significance. Mean values are given ± SEM. The regression model for factors influencing the Sholl profile included culture period/culture conditions, age and sex (1 = male, 2 = female) as explanatory variables.

## Results

**Figure 1:**
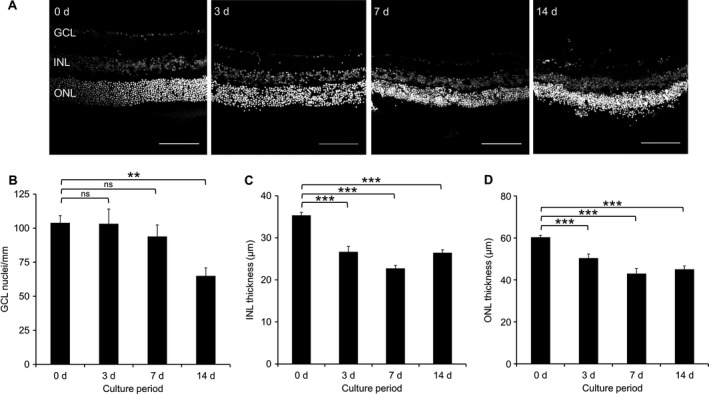
Nuclear stained frozen sections of explants cultured for up to 14 d. (A) Representative 8‐bit images of TO‐PRO‐3 labelled sections; scale bars 100 μm. (B) Linear cell counts of GCL show cell loss after 14 d. **P <* *0.005, ns, not significant, anova with Tukey post hoc. (C) INL thickness measured after each culture period show layer thinning after 3 d. ***P <* *0.001, anova with Tukey post hoc. (D) ONL thickness measured after each culture period show layer thinning after 3 d. ***P <* *0.001, Mann–Whitney with Bonferroni correction. *n *=* *5 retinas, 3 animals (0 d), *n *=* *6 retinas, 5 animals (3 d), *n *=* *3 retinas, 3 animals (7 d), *n *=* *4 retinas, 4 animals (14 d). At least three sections from each retina were analysed. Error bars ± SEM. d, days.

**Figure 2:**
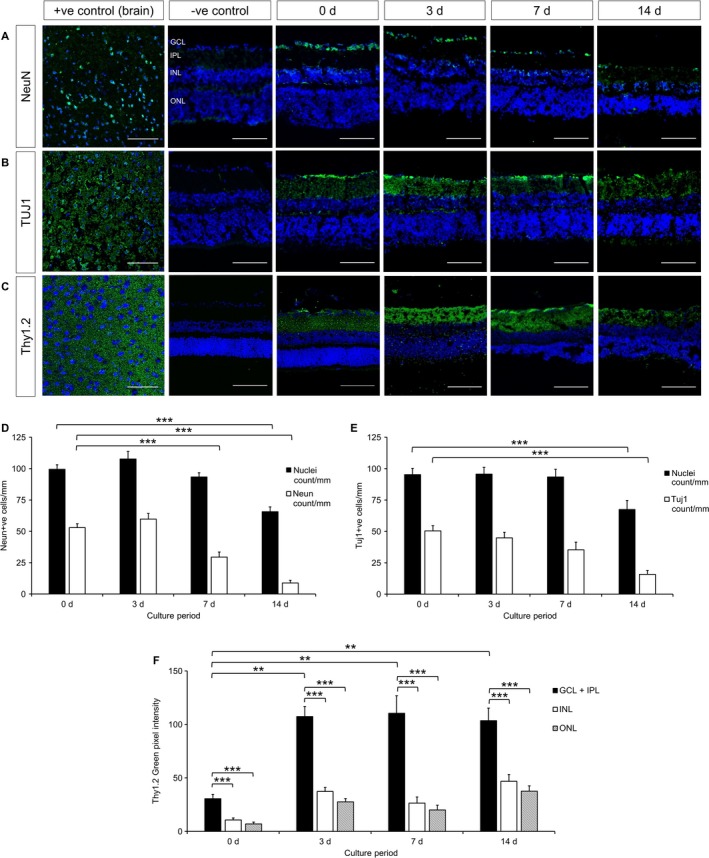
Immunofluorescence shows maintenance of neuronal markers over 14 d culture of retinal explants. (A) Staining for neuronal marker NeuN (green) counter‐stained with TO‐PRO‐3 nuclear stain (blue) in frozen sections of explants cultured for up to 14 d. *n *=* *3 retinas, 3 animals (0 d), *n *=* *3 retinas, 3 animals (3 d), *n *=* *3 retinas, 3 animals (7 d), *n *=* *3 retinas, 3 animals (14 d). (B) Staining for neuronal marker TUJ1 (green) counter‐stained with TO‐PRO‐3 nuclear stain (blue) in frozen sections of explants cultured for up to 14 d. *n *=* *3 retinas, 3 animals (0 d), *n *=* *3 retinas, 3 animals (3 d), *n *=* *3 retinas, 3 animals (7 d), *n *=* *3 retinas, 3 animals (14 d). (C) Staining for RGC marker Thy1.2 (green) counter‐stained with TO‐PRO‐3 nuclear stain (blue) in frozen sections of explants cultured for up to 14 d. *n *=* *3 retinas, 2 animals (0 d), *n *=* *3 retinas, 3 animals (3 d), *n *=* *3 retinas, 3 animals (7 d), *n *=* *5 retinas, 5 animals (14 d). (D) Quantification of NeuN‐positive cells in the GCL, shown with nuclear stained‐only cells for comparison. ***P <* *0.001, anova with Tukey post hoc. (E) Quantification of TUJ1‐positive cells in the GCL, shown with nuclear stained‐only cells for comparison. ***P <* *0.001, anova with Tukey post hoc. (F) Thy1.2 staining in each retinal layer quantified as mean green channel intensity, normalized for background fluorescence, as described in the text. **P <* *0.005, ***P <* *0.001, anova with Tukey post hoc. Positive controls: brain. Scale bars: 100 μm. At least three sections from each retina were analysed. Error bars ± SEM. d, days.

**Figure 3:**
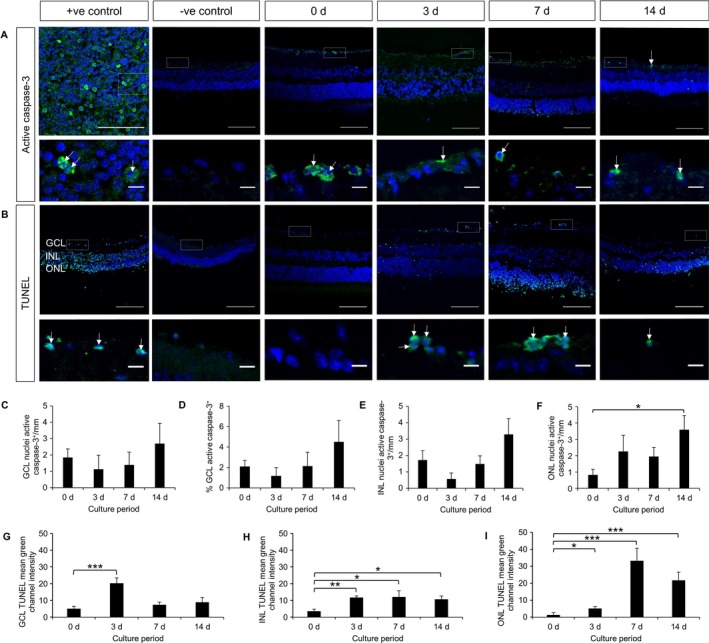
Fluorescent staining of apoptotic markers, active caspase‐3 and TUNEL in frozen sections of explants cultured for up to 14 d. (A) Active caspase‐3 staining (green) with TO‐PRO‐3 nuclear stain (blue) over 14 d at 20× magnification (top) and higher magnification of areas highlighted with dashed boxes to show individual cells (bottom). Active caspase‐3 staining tended to increase with time. Arrows indicate active caspase‐3 positive cells. Positive control: spleen. Scale bars: 100 μm (top), 10 μm (bottom). *n *=* *5 retinas, 3 animals (0 d); *n *=* *3 retinas, 3 animals (3 d); *n *=* *3 retinas, 3 animals (7 d); *n *=* *5 retinas, 5 animals (14 d). (B) TUNEL labelling (green) with TO‐PRO‐3 nuclear stain (blue) over 14 d at 20× magnification (top) and higher magnification of areas highlighted with dashed boxes to show individual cells (bottom). TUNEL labelling increased over time. Arrows indicate TUNEL‐positive cells. Positive control: proteinase‐k‐digested retinal sections. Scale bars: 100 μm (top), 10 μm (bottom). *n *=* *4 retinas, 3 animals (0 d); *n *=* *4 retinas, 4 animals (3 d); *n *=* *3 retinas, 3 animals (7 d); *n *=* *3 retinas, 3 animals (14 d). (C) Number of active caspase‐3‐positive cells in GCL quantified per mm. *P *>* *0.05, Mann–Whitney with Bonferroni correction. (D) Number of active caspase‐3‐positive cells in GCL quantified as % of cells in GCL. *P *>* *0.05, Mann–Whitney with Bonferroni correction. (E) Number of active caspase‐3‐positive cells in INL quantified per mm. *P *>* *0.05, Mann–Whitney with Bonferroni correction. (F) Number of active caspase‐3‐positive cells in ONL quantified per mm. *P <* *0.05, Mann–Whitney with Bonferroni correction. (G) TUNEL staining in GCL quantified as mean green channel fluorescence, normalized for background fluorescence. ***P <* *0.001, Mann–Whitney with Bonferroni correction. (H) TUNEL staining in INL quantified as mean green channel fluorescence, normalized for background fluorescence. *P <* *0.05, **P <* *0.005, Mann–Whitney with Bonferroni correction. (I) TUNEL staining in ONL quantified as mean green channel fluorescence, normalized for background fluorescence. *P <* *0.05, ***P <* *0.001, Mann–Whitney with Bonferroni correction. At least three sections from each retina were analysed. Error bars ± SEM. d, days.

Our first objective was to determine the time window during which dendrite extension can be studied before the death of axotomized RGCs sets in. The number of cells in the GCL, quantified by nuclear staining (Fig. 1A), was not found to decrease significantly during the first week following explantation but then did so by 2 weeks by 37.5% (*P *=* *0.001, anova with Tukey post hoc). The INL thickness decreased by 24.5% by 3 days (*P *=* *3.293 × 10^−11^, anova with Tukey post hoc), to a maximum of 35.6% by 7 days (*P *=* *3.815 × 10^−13^). The ONL thickness decreased by 16.4% after 3 days (*P *=* *1.18 × 10^−4^, Mann–Whitney tests with Bonferroni correction), to a maximum of 25.3% after 14 days (*P *=* *1.38 × 10^−11^, Fig. 1B). We next examined the expression of neuronal markers using antibodies to NeuN (Fig. 2A), TUJ1 (Fig. 2B) and Thy1.2 (Fig. 2C). During the same 2 week period the number of NeuN‐positive cells decreased by 83.5% (*P *=* *1.256 × 10^−6^, anova with Tukey post hoc, Fig. 2D), whereas the number of TUJ1‐positive cells decreased by 68.6% (*P *=* *3.408 × 10^−5^, anova with Tukey post hoc, Fig. 2E). In contrast, Thy1.2 staining increased 3.5‐fold after 3 days (*P *=* *0.002, anova with Tukey post hoc), and remained elevated thereafter (Fig. 2F). Next, cell death was examined by active caspase‐3 (Fig. 3A) or TUNEL staining (Fig. 3B). Active caspase‐3 staining was minimal in all layers at all time points, except in the ONL, where we measured a 4.4‐fold increase by 14 days (*P *=* *0.006, Mann–Whitney tests with Bonferroni correction, Fig. 3C–F). TUNEL staining was minimal in all layers at 0 day. Staining peaked at 3 days in the GCL (4.0‐fold increase, *P *=* *2.41 × 10^−4^, Mann–Whitney tests with Bonferroni correction), and at 7 days in the INL (3.5‐fold increase, *P *=* *6.93 × 10^−3^) and ONL (24.5‐fold increase, *P *=* *2.82 × 10^−70^), see Fig. 3G–I.

**Figure 4:**
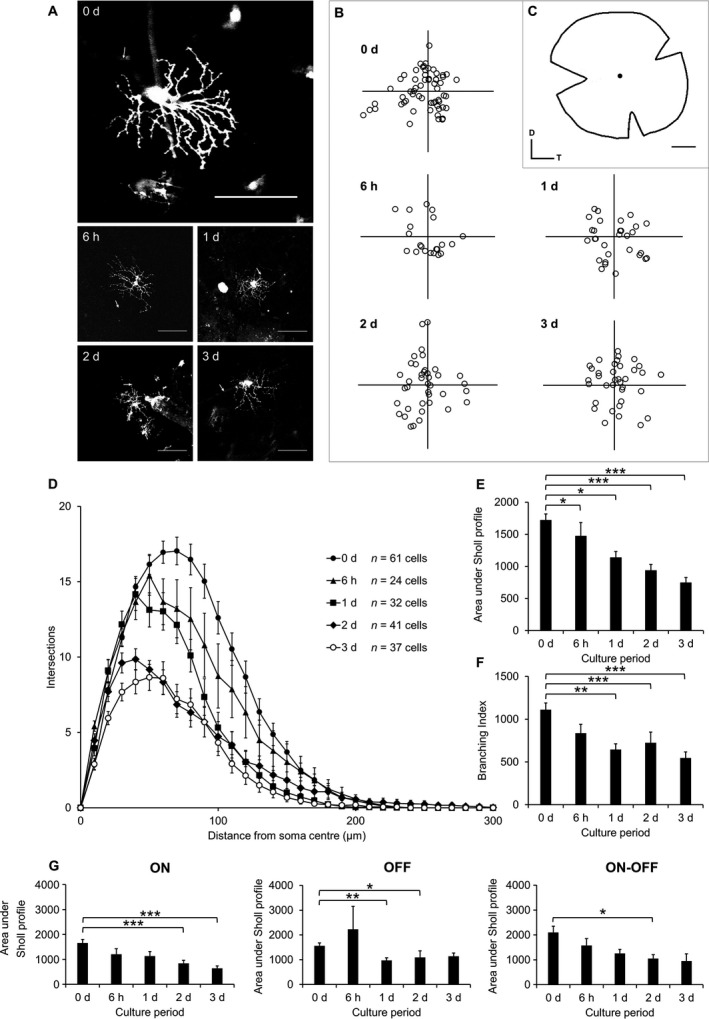
Diolistically labelled RGCs from explants cultured for up to 3 d show dendrite loss over time. (A) Representative 512 × 512 pixel (0 d) or 1024 × 1024 pixel (rest) images of RGCs at each time point; scale bars 100 μm; arrow indicates axon. (B) Locations of all labelled RGCs plotted relative to the optic nerve head (origin). (C) Diagram of prepared explant with optic nerve head indicated. D, dorsal; T, temporal; scale bar 1 mm. (D) Sholl plots for RGCs at each time point. (E) Area under Sholl profiles at each time point. *P <* *0.05, ***P <* *0.001, Mann–Whitney with Bonferroni correction. (F) Branching index of RGCs. **P <* *0.005, ***P <* *0.001, anova with Tukey post hoc. (G) Sholl AUC for each culture period split by RGC sub‐type. ON (left): *n* = 28 cells (0 d), *n* = 14 cells (6 h), *n* = 13 cells (1 d), *n* = 26 cells (2 d), *n* = 28 cells (3 d). OFF (middle): *n* = 12 cells (0 d), *n* = 4 cells (6 h), *n* = 8 cells (1 d), *n* = 8 cells (2 d), *n* = 5 cells (3 d). ON‐OFF (right): *n* = 11 cells (0 d), *n* = 6 cells (6 h), *n* = 11 cells (1 d), *n* = 7 cells (2 d), *n* = 4 cells (3 d). *P <* *0.05, **P <* *0.005, ***P <* *0.001, Mann–Whitney with Bonferroni correction. The numbers of cells analysed (D) are indicated. Error bars ± SEM. d, days.

Having established that no significant signs of cell death is observed in the GCL during the first week of culture, RGCs were labelled diolistically for morphometric analysis (Fig. 4A–C). Surprisingly, dendritic shrinkage, measured as a decrease in Sholl area under the curve (AUC), could be observed as early as after 6 h post‐mortem (*P *=* *4.91 × 10^−2^, Mann–Whitney tests with Bonferroni correction, Fig. 4A–C), which further progressed over the next 3 days (*P *=* *2.42 × 10^−9^, Fig. 4D and E). The branching index reduced by 41.9 ± 6.0% by 1 day (*P *=* *0.007, anova with Tukey post hoc), after which it remained relatively unchanged (Fig. 4F). Having defined a window of several days during which alterations in the dendritic profile could be analysed in the absence of cell death, the Sholl profiles were also determined separately for ON, OFF, bistratified RGC sub‐types. No bias could be detected at any of the time points examined. Thus, compared with the same cell type at time 0, the Sholl AUC of ON cells decreased by 49.0 ± 7.1% (*P *=* *4.02 × 10^−4^, Mann–Whitney tests with Bonferroni correction) after 2 days and by 61.0 ± 5.6% (*P *=* *2.50 × 10^−5^) after 3 days. The corresponding values for the OFF cells decreased by 37.9 ± 6.5% (*P *=* *1.91 × 10^−3^) after 1 days and by 30.1 ± 17.0% (*P *=* *4.96 × 10^−2^) after 2 days and for the bistratified cells 48.0 ± 8.1% (*P *=* *4.83 × 10^−2^) after 2 days (see also Fig. 4G). To examine any potential role of confounding variables, a multiple regression analysis was run with Sholl AUC as the dependent variable and culture period, age and sex as predictors. The model provided a good fit for the data (*P *=* *7.27 × 10^−12^, anova) with the time of culture period as the only significant predictor (B = − 228, *P *=* *7.08 × 10^−10^) of Sholl AUC.

**Figure 5:**
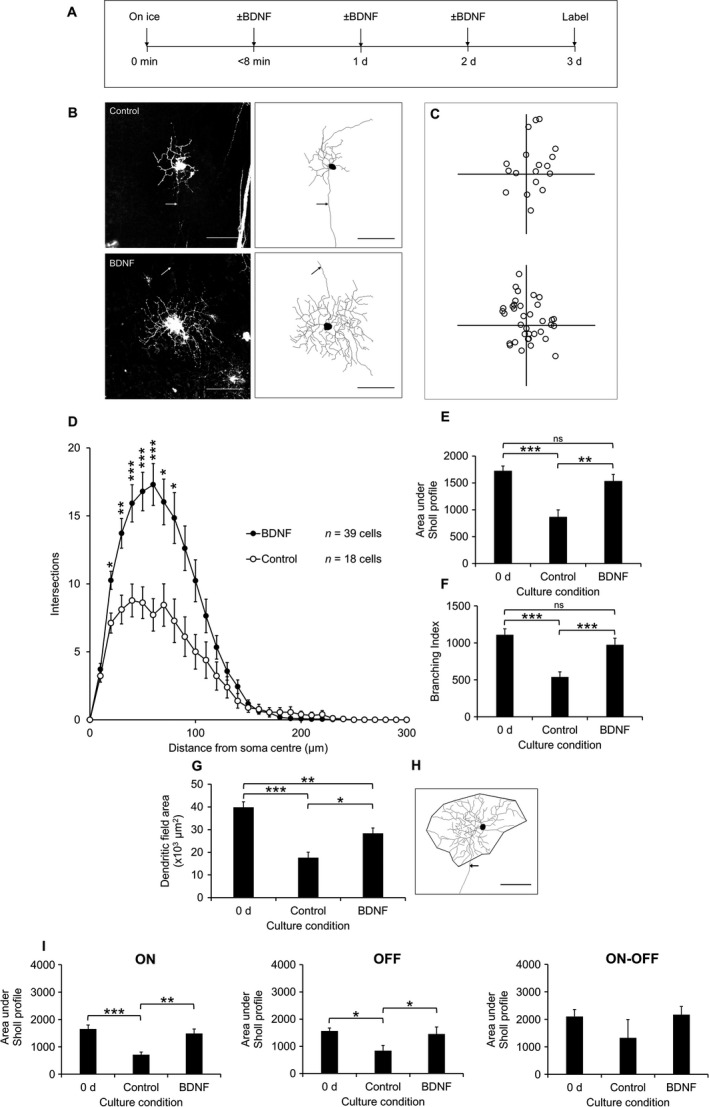
Diolistically labelled RGCs from explants cultured with 100 ng/mL BDNF, or vehicle (PBS, 0.1% BSA) as control, over 3 d showing retardation of dendritic atrophy in the presence of BDNF. (A) Time course of experiment. (B) Representative 1024 × 1024 pixel images of RGCs from control (top) and BDNF (bottom) with 8‐bit tracing images for each cell (right); scale bars 100 μm; arrow indicates axon. (C) Locations of RGCs from control (top) and BDNF‐treated (bottom) explants plotted relative to the optic nerve head (origin). (D) Sholl profiles for RGCs in each group. *P <* *0.05, **P <* *0.005, ***P <* *0.001, Kruskal–Wallis. (E) Area under Sholl profiles in each group, shown with the 0 d value for comparison. **P <* *0.005, ***P <* *0.001, ns, not significant, anova with Tukey post hoc. (F) Branching index. ***P <* *0.001, ns, not significant, anova with Tukey post hoc. (G) Dendritic field area of RGCs, shown with the 0 d value for comparison. *P <* *0.05, **P <* *0.005, ***P <* *0.001, anova with Tukey post hoc. (H) Example of dendritic field area measurement using 8‐bit tracing. Scale bar 100 μm, arrow indicates axon (not included in measurement). (I) Sholl AUCs split according to RGC stratification depth. ON (left): *n* = 28 cells (0 d), *n* = 8 cells (control), *n* = 26 cells (BDNF). OFF (middle): *n* = 20 cells (0 d), *n* = 7 cells (control), *n* = 10 cells (BDNF). ON‐OFF (right): *n* = 13 cells (0 d), *n* = 3 cells (control), *n* = 3 cells (BDNF). *P <* *0.05, **P <* *0.005, ***P <* *0.001, anova with Tukey post hoc. The number of cells analysed (D) are indicated. Error bars ± SEM. d, days.

Having established that our culture conditions and labelling technique allow us to analyse the role of BDNF in modulating dendritic atrophy following axotomy without the confounding factor of RGC loss or of biases towards particular RGC populations, we next supplemented the culture medium for a period of 3 days with BDNF added at 0 or 3 days (see Fig. 5A for a summary of the procedure, with representative cells illustrated in Fig. 5B and C). Following incubation with BDNF, the Sholl profile increased at 20–80 μm from the soma centre (*P *<* *0.05, Kruskal–Wallis), relative to controls (Fig. 5D). The Sholl AUC increased by 75.8 ± 14.8% (*P *=* *0.002, anova with Tukey post hoc) relative to controls and was not significantly different to that measured at 0 day (Fig. 5E). The addition of BDNF increased branching by 81.0 ± 15.7% (*P *=* *1.94 × 10^−4^, anova with Tukey post hoc), relative to controls and was not significantly different compared to 0 day cells (Fig. 5F). The dendritic field area of BDNF‐treated cells was 61.2 ± 13.9% (*P *=* *0.011, anova with Tukey post hoc) larger compared with controls and 28.8 ± 6.1% (*P *=* *0.004) smaller than that of 0 day cells (Fig. 5G and H). We next examined possible sub‐type‐specific effects of BDNF and analysed ON, OFF and ON‐OFF RGCs based on IPL stratification depth. The Sholl AUC of BDNF‐treated ON cells increased by 108.9 ± 22.4% (*P *=* *0.003, anova with Tukey post hoc) relative to controls, not significantly different from 0 day cells. BDNF‐treated OFF cells increased by 73.6 ± 30.2% (*P *=* *0.047) relative to controls, with no significant difference from 0 day cells, as was also the case for bistratified cells (Fig. 5I). The possible confounding effects of age and sex were tested by multiple regression analysis. Area under the Sholl profile was the dependent variable and culture condition (1 = control and 2 = BDNF‐treated), age and sex were predictors. The model provided a good fit of the data (*P *=* *0.022, anova) and in this case, the culture condition, plus or minus BDNF, was found to be the only significant predictor (B = 665, *P *=* *0.008) of Sholl AUC.

**Figure 6:**
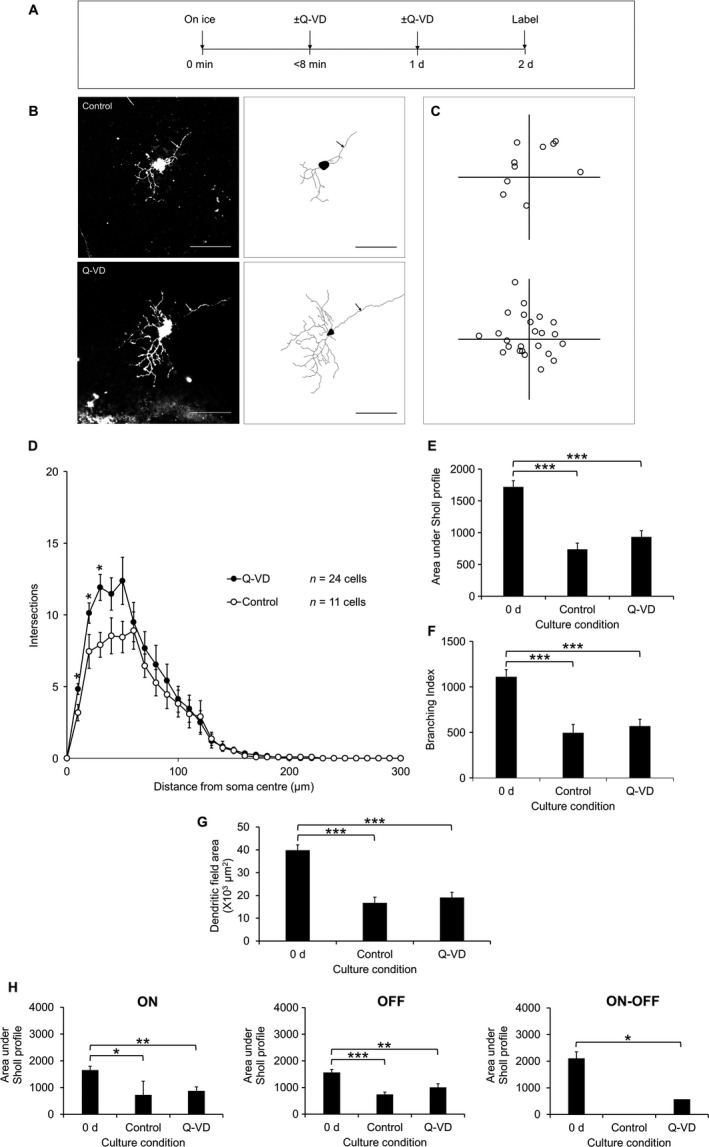
Diolistically labelled RGCs from explants cultured with 100 μm pan‐caspase inhibitor, Q‐VD, or vehicle (DMSO) as control over 2 d showing modest protective effect of Q‐VD on dendritic atrophy of RGCs. (A) Time course of experiment. (B) 1024 × 1024 pixel images of fluorescently labelled RGCs from control (top) and Q‐VD‐treated (bottom) explants with 8‐bit tracing images for each cell (right). Scale bars 100 μm. Arrow indicates axon. (C) Locations of every labelled RGC from control (top) and Q‐VD‐treated (bottom) explants plotted relative to the optic nerve head (origin). (D) Sholl profiles of labelled RGCs. *P <* *0.05, anova. (E) Area under Sholl profiles shown with the value for 0 d for comparison. ***P <* *0.001, anova with Tukey post hoc. (F) Branching index of RGCs. ***P <* *0.001, anova with Tukey post hoc. (G) Dendritic field area of RGCs in each group shown with the value for 0 d as comparison. ***P <* *0.001, anova with Tukey post hoc. (H) Sholl AUCs split by RGC sub‐type. ON (left): *n* = 28 cells (0 d), *n* = 2 cells (control), *n* = 10 cells (Q‐VD). OFF (middle): *n* = 12 cells (0 d), *n* = 9 cells (control), *n* = 13 cells (Q‐VD). ON‐OFF (right): *n* = 11 cells (0 d), *n* = 0 cells (control), *n* = 1 cell (Q‐VD). *P <* *0.05, **P <* *0.005, ***P <* *0.001, anova with Tukey post hoc. The numbers of cells analysed (D) are indicated. Error bars ± SEM. d, days.

We next determined the extent to which dendritic degeneration could be mitigated by caspase inhibition within 2 days of culture (Fig. 6A–C), a time interval selected as this presented the peak for the change in dendrite configuration. Indeed, recent results suggest that the degeneration of neuronal processes may involve mechanisms requiring caspase activity (Williams *et al*., 2006; Nikolaev *et al*., 2009; D'Amelio *et al*., 2010; Hyman & Yuan, 2012; Erturk *et al*., 2014). While the Sholl profile was greater at 10–30 μm compared with untreated controls (*P *<* *0.05, Kruskal–Wallis, Fig. 6D), the Sholl AUC, branching index and dendritic field area while showing a similar trend, did not reach significance (Fig. 6E–G). Given this trend and the possibility of cell selectivity in the effect of Q‐VD, RGC sub‐type‐specificity was also examined with ON, OFF and ON‐OFF separately examined. However, this analysis did not reveal any evidence of a differential protective effect of pan‐caspase inhibitors. Age was also checked as a possible confounder by multiple regression analysis but again the only variable found to be a predictor of Sholl AUC was the presence or absence of Q‐VD in the culture medium (B = 464, *P *=* *0.005).

**Figure 7:**
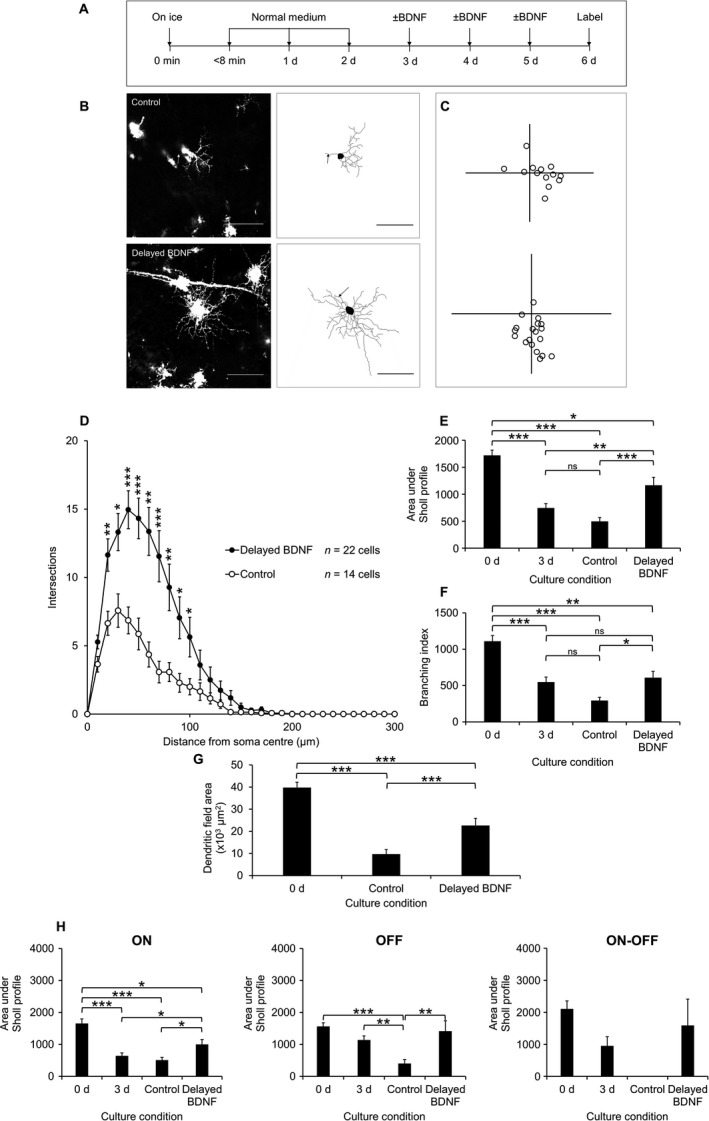
Diolistically labelled RGCs from explants cultured with 100 ng/mL BDNF or vehicle (PBS, 0.1% BSA) as control over 3 d from day 3 showing that delayed BDNF treatment is capable of retarding dendritic retraction of RGCs. (A) Time course of experiment. (B) Representative 1024 × 1024 pixel images of RGCs from control (top) and delayed BDNF‐treated (bottom) explants with 8‐bit tracing images for each cell (right); scale bars 100 μm; arrow indicates axon. (C) Locations of RGCs from control (top) and delayed BDNF‐treated (bottom) explants plotted relative to the optic nerve head (origin). (D) Sholl profiles for RGCs. *P <* *0.05, **P <* *0.005, ***P <* *0.001, Kruskal–Wallis. (E) Area under Sholl profiles in each group, shown with the values for 0 and 3 d for comparison. *P <* *0.05, **P <* *0.005, ***P <* *0.001, ns, not significant, anova with Tukey post hoc. (F) Branching index of RGCs. *P <* *0.05, **P <* *0.005, ***P <* *0.001, ns, not significant, anova with Tukey post hoc. (G) Dendritic field area of RGCs shown with value for 0 d as comparison. **P <* *0.005, anova with Tukey post hoc. (H) Sholl AUCs split according to RGC sub‐type. ON (left): *n* = 28 cells (0 d), *n* = 12 cells (control), *n* = 14 cells (delayed BDNF). OFF (middle): *n* = 12 cells (0 d), *n* = 2 cells (control), *n* = 6 cells (delayed BDNF). ON‐OFF (right): *n* = 11 cells (0 d), *n* = 0 cells (control), *n* = 2 cells (delayed BDNF). *P <* *0.05, **P <* *0.005, ***P <* *0.001, ns, not significant, anova with Tukey post hoc. The number of cells analysed (D) are indicated. Error bars ± SEM. d, days.

Given the potential clinical relevance of the finding that BDNF retards the progression of dendritic atrophy, we next examined whether its delayed addition would also be effective, given the clear progression of dendritic retraction following axotomy in the absence of RGC death (see Fig. 7A–C). The corresponding Sholl profile was greater at 20–100 μm (*P *<* *0.05, Kruskal–Wallis) compared with untreated controls (Fig. 7D). Sholl AUC increased by 135.6 ± 29.6% (*P *=* *1.08 × 10^−4^, anova with Tukey post hoc) relative to controls and 56.6 ± 19.7% (*P *=* *0.003) relative to 3 days, but decreased by 32.2 ± 8.5% (*P *=* *0.009) relative to 0 day (Fig. 7E). The branching index for delayed BDNF‐treated cells was increased by 106.8 ± 29.2% (*P *=* *0.009, anova with Tukey post hoc), compared to controls, but decreased by 45.1 ± 7.7% relative to 0 day (*P *=* *0.002), relative to 0 day (Fig. 7F). The dendritic field area was 133.9 ± 32.8% increased (*P *=* *1.93 × 10^−5^, anova with Tukey post hoc), compared with untreated controls, but was 43.1 ± 8.0% decreased (*P *=* *4.59 × 10^−5^), compared to 0 day (Fig. 7G). To check for sub‐type‐specific effects of delayed BDNF treatment, ON, OFF and ON‐OFF cells were also examined separately. The Sholl AUC for ON cells was increased by 95.8 ± 30.4% (*P *=* *0.020, anova with Tukey post hoc) relative to untreated cells and increased by 55.2 ± 24.1% (*P *=* *0.025) relative to 3 days cells, and 39.5 ± 9.4% decreased (*P *=* *0.033), compared with 0 day cells. The Sholl AUC for OFF cells was increased by 253.3 ± 80.2% (*P *=* *0.001) compared with untreated cells, though this was not significantly different compared with 0 and 3 days cells. The Sholl AUC of bistratified cells was not significantly different between groups (Fig. 7H). There was no significant difference for BDNF‐treated cells between sub‐types, indicating an absence of sub‐type‐specific effects of BDNF. Regression analysis confirmed culture condition (1 = control, 2 = delayed BDNF‐treated) as the only factor influencing dendritic integrity (B = 671, *P *=* *0.001).

## Discussion

The key novel finding here is that *delayed* application of BDNF significantly retards dendritic atrophy of RGCs following retinal explantation. While this manuscript was being revised, a related study was published using mouse retinal explants and time‐series imaging of Thy1‐YFP‐labelled RGCs (Johnson *et al*., 2016). This study showed that the joint addition of ciliary neurotrophic factor and BDNF at the time of explantation attenuates dendritic loss over the effects of forskolin alone, thus broadly supporting our conclusions (see below for further discussion).

To ensure that under our experimental conditions, the loss of dendrites can be studied in the absence of RGC death, we first established the time course of cell loss in the GCL. We found that the pattern of cell loss identified by the RGC markers NeuN, Tuj1 and Thy1.2 was inconsistent, likely reflecting the lack of truly specific markers (Perry *et al*., 1984; Gan *et al*., 1996). In addition, the expression of proteins, including Thy1 (Lee *et al*., 1998; Schlamp *et al*., 2001; Huang *et al*., 2006; Astafurov *et al*., 2014), may alter following injury, as we report here (Fig. 2). Therefore, we concluded that the use of nuclear staining to quantify RGC loss is more reliable, given that amacrine cells are resistant to degeneration (Kunzevitzky *et al*., 2010) and that the numbers of apoptotic cells in the GCL detected by TUNEL and active caspase‐3 were negligible over 14 days. Using nuclear staining, we found that RGC counts do not decrease significantly during the course of the first week (Fig. 1B). As the loss of RGC dendrites can already be observed 6 h after explantation (Fig. 4D), a substantial time window exists during which the protective effects of BDNF can be studied, even under conditions of delayed addition (Figs 5 and 7). A detailed analysis of the process of dendritic retraction (Fig. 4D) revealed an initial leftward shift of the (Sholl) profile, possibly resulting from selective loss of distal dendrites, as reported in axotomized mouse RGCs (Morquette *et al*., 2015). In contrast, we can discount sub‐type bias after finding no significant differences in Sholl AUC between ON, OFF or bistratified cells at any time point, and the absence of sampling bias as a function of eccentricity (Fig. 4B). A key difference between our study and the results published by Johnson *et al*., 2016 lies in the method used to assess dendritic arbourization. While we randomly labelled cells by diolistics at different time points, Johnson *et al*. use a Thy‐1 YFP line that also sparsely labelled RGCs, with the advantage that individual cells can be followed over time. However, in addition to the persistent unresolved question of the mechanisms leading to single‐cell marking that may or may not select for subclasses of neurons in the CNS, we previously noted that genetically encoded reporters such as YFP can underreport dendritic pathology (Williams *et al*., 2013), possibly because probes are more likely to be expressed in cells less affected by the lesion, with a biosynthetic machinery that is still intact. In contrast, diolistics labelling relies on temperature‐dependent diffusion within the plasma membrane, regardless of the health of the cell. This may explain why in the very recent study by Johnson and colleagues, dendritic pathology was not reported before 7 days of culture, a time point at which the death of RGCs was already significant (Johnson *et al*., 2016). This temporal coincidence makes it difficult to separate cell death from dendritic atrophy and while we have no explanation as to why under our experimental conditions, no significant RGC death (determined by nuclear staining) can be observed during the first week following explantation, we think that our much earlier detection of dendritic atrophy may be explained by a combination of the use of an unbiased labelling method (Sherazee & Alvarez, 2013) coupled with the underreporting dendritic pruning when using Thy1‐YFP animals (Williams *et al*., 2013).

As such, the effects of BDNF on dendrites may not be too surprising given previous reports by others in various areas of the CNS, typically during development (McAllister *et al*., 1995, 1996; Bosco & Linden, 1999; Horch *et al*., 1999; Lom & Cohen‐Cory, 1999; Jin *et al*., 2003; Baquet *et al*., 2004; Rauskolb *et al*., 2010). Of special relevance are previous experiments indicating that BDNF treatment reduces dendritic retraction of RGCs *in vivo* in a cat optic nerve crush model (Weber & Harman, 2008) and an optic nerve transection model (Rodger *et al*., 2012).

In conclusion, we show that application of BDNF alone attenuates dendritic retraction of randomly labelled RGCs in the adult retinal explant. Crucially, this BDNF protective effect can also be observed following delayed application of BDNF, a significant new observation that may have important implications for the treatment of neurodegenerative disorders, such as glaucoma.

## Conflict of interests

The authors have no conflict of interest to declare.
